# A New, Better BET: Rescuing and Revising Basic Emotion Theory

**DOI:** 10.3389/fpsyg.2018.01217

**Published:** 2018-07-17

**Authors:** Daniel D. Hutto, Ian Robertson, Michael D. Kirchhoff

**Affiliations:** Department of Philosophy, Faculty of Law, Humanities and the Arts, University of Wollongong, Wollongong, NSW, Australia

**Keywords:** basic emotions, affect programs, affective science, embodiment, radical enactivism

## Abstract

Basic Emotion Theory, or BET, has dominated the affective sciences for decades (Ekman, 1972, 1992, 1999; Ekman and Davidson, 1994; Griffiths, 2013; Scarantino and Griffiths, 2011). It has been highly influential, driving a number of empirical lines of research (e.g., in the context of facial expression detection, neuroimaging studies and evolutionary psychology). Nevertheless, BET has been criticized by philosophers, leading to calls for it to be jettisoned entirely (Colombetti, 2014; Hufendiek, 2016). This paper defuses those criticisms. In addition, it shows that we have good reason to retain BET. Finally, it reviews and puts to rest worries that BET’s commitment to affect programs renders it outmoded. We propose that, with minor adjustments, BET can avoid such criticisms when conceived under a radically enactive account of emotions. Thus, rather than leaving BET behind, we show how its basic ideas can be revised, refashioned and preserved. Hence, we conclude, our new BET is still a good bet.

“I don’t experience basic human emotions. It’s not my thing”

- Albert Brooks

## Introduction

Basic Emotion Theory, or BET, conjectures that at least some emotions can be identified with or otherwise instantiate biologically evolved, pan-cultural affect programs (Ekman, 1972; Griffiths, 1997; DeLancey, 2002).^1^ Understood as affect programs, basic emotions unfold, without conscious attention or direction, in coordinated yet automatic, script-like patterns – where such patterns manifest as a distinctive mosaic of physiological, autonomic, neural, behavioral, and expressive organismic responses^2^.

Basic Emotion Theory has had a good run. It has been influential in shaping research in the affective sciences for decades (Ekman, 1992, 1999; Ekman and Davidson, 1994; Scarantino and Griffiths, 2011). It has opened up new lines of research in psychology – on the detection of facial expressions; in neuroimaging – on the neural correlates of basic emotions; and in evolutionary psychology^3^. Nevertheless, philosophers have criticized BET on both methodological and theoretical grounds. This has led to calls for BET to be jettisoned entirely (Colombetti, 2014; Hufendiek, 2016).

The structure of this paper is as follows. Section “All BETs Are Off!” outlines the objections that Colombetti (2014) and Hufendiek (2016) raise against BET. These objections have led them to recommend either avoiding BET or rejecting it wholesale. Section “Is BET Really a Bad BET?” defuses the criticisms leveled at BET by showing that they rest on drawing the basic/non-basic distinction in just one possible way – which is not the best motivated way to draw the distinction. Section “Reasons to BET On BET” shows that, once that distinction is understood in terms of non-basic emotions having an additional ingredient, it is possible to provide a plausible account of how non-basic emotions arise and how they relate to basic emotions. Finally, Section “Adjusting our BET” shows that BET is not harmed by its commitment to affect programs, since these need neither be conceived of as brain-bound sets of instructions nor as inflexible, hardwired, automatic, stimulus-response patterns. Rather than leaving BET behind, we show how its basic ideas can be revised, refashioned and rescued by appeal to a radically enactive view of the emotions. All in all, we propose that our new, adjusted BET is still a very good bet^4^.

## All Bets Are Off!

Why bet against BET? In a critical review of the state of emotion research, Colombetti (2014) identifies a series of objections that have been leveled against BET. These include objections that BET is to be avoided because it is: (1) refuted by cross-cultural linguistic differences; (2) tainted by use of a flawed, forced choice methodology in collecting evidence for pan-cultural facial expressions; (3) incapable of accommodating the context-sensitivity and openness of emotional episodes; (4) unsupported by contemporary neuroimaging data (for full details and discussion, see Colombetti, 2014, p. 29–36).

In the end, none of these concerns prove fatal. Indeed, Colombetti (2014) does excellent work in exposing each of these objections to be misguided or otherwise ineffectual. Nevertheless, Colombetti (2014) and Hufendiek (2016) have identified other problems with the distinction between basic and non-basic emotions that these critics hold hit BET, and hit it hard. Indeed, in betting against BET these authors take their complaints to be serious enough to constitute independently sufficient reasons for steering clear of BET, if not for rejecting it outright.

Colombetti (2014) maintains that the most serious problem with BET is that its prominence in affective science has led researchers to the counterproductive conviction that emotions divide into two distinct, mutually exclusive classes on the assumption that basic and non-basic emotions can share no class-defining properties in common. Fundamentally, BET has thereby promoted belief in a distorting taxonomy according to which some emotions are deemed to possess distinctive pan-cultural response profiles while others are deemed to be entirely culture-bound and lacking distinctive response profiles (Colombetti, 2014, p. 36).

How has this come about? On Colombetti’s (2014) analysis, the belief that we have already identified some basic emotions has given credence to the idea that BET’s basic/non-basic emotion distinction is empirically well-established. That idea, in turn, fuels blinkered research programs that perpetuate the unfounded belief in the basic/non-basic distinction itself because they spuriously focus on only a subset of emotions. In effect, unprincipled decisions about which emotions should be investigated as candidate basic emotions have made it seem, illogically, as if we have evidence that only some emotions are basic.

Ultimately, Colombetti (2014) traces the source of the trouble to the influence of the seminal study by Ekman and Friesen (1971) – a study that first helped launch BET into the limelight. Ekman and Friesen (1971) sought to determine whether the same facial expressions would be identified as displaying the same emotions in both literate and non-literate cultures. Their famous cross-cultural study of Westernized and non-Westernized subjects revealed that both populations associated a select set of facial patterns with a particular set of emotions, namely: anger, disgust, fear, happiness, surprise, and sadness. These findings have been widely taken to support the idea that the links between some facial expressions and some emotions may indeed be universal. These findings led, initially, to the conviction that there are, at least, six basic emotions^5^.

Importantly, the process by which these six emotions made the basic emotion short list was arbitrary. As Colombetti (2014, p. 38) reminds us, the emotions in the Ekman and Friesen study were not chosen “on the basis of a clear rationale.” In fact, merely practical restrictions drove Ekman and Friesen (1971) to focus on the six emotions they did, rather than a wider array of candidates^6^. Basically, they choose the six emotions in question because they could not obtain enough suitable sample photographs to investigate a broader set. Naturally, deciding which emotions are basic by such a selection process is clearly arbitrary.

Colombetti (2014) acknowledges that Ekman and Friesen (1971) never claimed or intended their list of six emotions to be a principled or exhaustive list of the basic emotions. Nevertheless, she takes it to be a bad consequence of the influence of their early findings that emotion researchers have come to assume, rather than empirically establish, that there is a distinctive class of basic emotions. With respect to the arbitrariness of the list of candidate basic emotions things have not improved. This is so, even though the list of basic emotions waxes and wanes and need not be exactly the same as the six identified in the Ekman and Friesen (1971) study^7^.

Putting all of this together, the way BET has been taken up in the field –precisely because of its great influence– is doing more harm than good. In particular, Colombetti (2014) maintains that BET is a self-perpetuating and distorting theoretical framework. The assumption that some emotions are basic and others are not has led to those deemed non-basic being less vigorously studied. Worse, this has led to a dearth of evidence for assessing whether emotions deemed non-basic are, in fact, non-basic. For example, “the conviction that emotions such as jealousy, shame, envy, love, and so on are not basic and thus do not have distinctive manifestations has discouraged the study of their neural, behavioral and bodily features” (Colombetti, 2014, p. 40, see also Shaver et al., 1996). As such, she holds that BET is not empirically well-established so much as widely presupposed.

Notably, even those who do not embrace BET or who are skeptical of it can still shape their research agendas in light of it. Nor does it matter if BET’s progenitors decry this situation or not. For, if Colombetti (2014, p. 37) is right, “BET has acquired a life of its own, and the received view in affective science today is that only some emotions are basic.” Her reasoning is straightforward: Only an arbitrary sub-class –not the full class– of emotions is systematically investigated to see whether they qualify as basic. If so, then the supposition that *only some* emotions are basic is empirically unfounded and has irrationally restricted the focus of affective science. Because of this assessment of BET, she maintains that affective scientists should dispense with it and the notion of basic emotions entirely^8^.

Hufendiek (2016) also rails against BET. She questions the conceptual tenability and evidential basis of BET’s basic/non-basic emotion distinction, as she construes it. Like Colombetti (2014), she assumes that BET is committed to “*the neat distinction* between basic emotions, which are evolutionarily acquired affect programs, and higher cognitive emotions, which do not have a unique pattern of bodily reactions... but presuppose higher cognitive abilities” (p. 66, emphasis added).

Hufendiek takes Griffiths (1997) to be the official spokesperson for BET on the question of the basic/non-basic emotion distinction. This leads her to conclude that BET is committed to the idea that “basic emotions form a natural kind that was already present in our ancestors, [and that] higher cognitive emotions should be seen as *a distinct class*” (Hufendiek, 2016, p. 65, emphasis added). Yet, though she does not state it explicitly, her assumption is stronger. For, in formulating the basic/non-basic emotion distinction Griffiths-style, any given emotion must fall into one of two mutually exclusive, distinct classes on the assumption that the two kinds of emotions can share no properties in common.

Basic Emotion Theory is thus taken to subscribe to an anti-essentialist position about non-basic emotions such that they are “cognitive-based and culturally constructed phenomena, and not natural kinds” (Caruana, 2017, p. 87). It is in this sense that BET is purportedly driven to assume that non-basic emotions are, as Hufendiek (2016, p.66) puts it, “without any roots in … evolutionary history.” For this reason, emotions of these different kinds are assumed to depend on entirely different sorts of mechanisms. Non-basic emotions are marked out as lacking the features of affect programs precisely because they have not been evolutionary acquired. This is what allegedly makes non-basic emotions “completely different from basic ones” (Caruana, 2017, p. 88).

Accordingly, if the preceding analysis is correct, BET embeds a disunity thesis – one that firmly distinguishes basic from non-basic emotions. Bearing all this in mind, Hufendiek’s (2016, p. 66) argument against BET is based on her analysis that “there is no reason for the radical distinction drawn by Griffiths.”

Her complaints on this score vary in content, scope and strength. She tells us that dividing basic and non-basic emotions into two distinct and opposing classes “does not make much sense” (Hufendiek, 2016, p. 67); or that at the very least such a division is “much too strict” (Hufendiek, 2016, p. 66). However, she does not give any persuasive arguments about how much sense the distinction actually makes or the degree of strictness it enjoys. Rather, her true focus seems to be on whether it is warranted at all and her predictions about its empirical scope and applicability.

With respect to the question of warrant, she thinks the basic/non-basic emotion distinction only looks attractive because we fail to see the woods for the trees. She holds the distinction is not justified because it reads too much into the evidence gleaned from a limited set of scientific findings. There is positive data that she takes to supply evidential support for the proposition “that *all* emotions, not simply basic emotions, possess stereotypical physiological, behavioral, and expressive characteristics” (Hufendiek, 2016, p. 66, emphasis added). If that were so, then all emotions would exhibit features of affect programs.

What evidential support is there for such a conclusion? Hufendiek (2016) provides instances in which purportedly paradigmatic non-basic emotions exhibit affect program features. She points to shame and pride as principal examples. She cites a range of findings that are consistent with the possibility that many paradigmatic non-basic emotions may, after all, have a hardwired neurological profile (Fessler, 2004; Tracy and Robbins, 2004, 2007a,b; Beer, 2007; Tracy et al., 2013). Furthermore, she speculates that “since research in this field is still very young … we can expect similar evidence for many, if not all [non-basic] emotions” (Hufendiek, 2016, p. 66)^9^.

Putting all of this together, ultimately, she concludes that “a *strong case* can … be made for the unity thesis that emotions do belong to the same natural kind” (Hufendiek, 2016, p. 66, emphasis added). That, of course, would be to go too far. For example, at this stage of the empirical game, it is only fair to say that we do not know that shame and pride lack a “dedicated neural circuitry” (Hufendiek, 2016, p. 67). In line with this more judicious reading of the evidence, in places Hufendiek (2016) is more cautious and guarded. Sometimes her argument against BET only amounts to the observation that the positive evidence for an absolute basic/non-basic emotion distinction is “pretty thin” (Hufendiek, 2016, p. 67). Her more careful, and credible, assessment is “that there *might be* basic forms … for far more emotions” (Hufendiek, 2016, p. 65, emphasis added).

Empirically speaking, it seems that there is only one reasonable answer to the question of whether the basic/non-basic emotion distinction holds up: namely, it is “considerably too early to conclude” (Hufendiek, 2016, p. 67). In the end, this more careful assessment of the sum total of the current empirical data cannot support a recommendation, here-and-now, that affective science is better off without the idea of basic emotions.

## Is Bet Really a Bad Bet?

As we have just seen, some recommend getting rid of or at least betting against BET (Colombetti, 2014; Hufendiek, 2016). But before we rush to judgment, we should ask: Do their arguments –even when cast into their ideal, perfected forms– hold up?

Let us first return to Colombetti’s (2014) worries and start by considering the factual question of whether or not the basic/non-basic distinction actually has the pernicious, self-perpetuating effects that she ascribes to it. Is it really the case that affective science, impaired by BET’s distinction, has overly restricted the scope of inquiries?

Nearly two decades ago, Shaver et al. (1996) anticipated Colombetti’s (2014) observations about BET’s supposedly bad effects. Using love as the principle example, they claimed that the fact that the leading theorists of the day left love off their lists of basic emotions resulted in a virtually loveless affective science – one in which love received “less attention from emotion researchers than its place in everyday life would lead one to expect” (p. 3).

Perhaps love was a neglected target of investigation 20 years ago. But even if this were so then, it would be hard to argue that the same holds true of today’s affective science. For example, Graham (2011) provides a meta-analysis of higher-order factors underlying the most popular measures of love, reviewing 81 studies that analyze data from 103 samples and 19,387 individuals (see also Ortique et al., 2010; Xu et al., 2011).

Or, consider the body of evidence that Hufendiek (2016) cites in making her case against BET. Recall the range of studies concerning pride and shame. Taken at face value, contra Colombetti’s (2014) worry that BET is imposing a narrow focus on the field, if anything, it seems that the actual trend appears to be going in the opposite direction. Emotions that are canonically non-basic, or at least which straddle the basic/non-basic line, are being regularly investigated to see whether they exhibit basic profiles.

Perhaps this is hardly surprising, for even as far back as 1992 (Ekman, 1992) disavowed that basic emotions need to be restricted to a small sub-set. He openly entertained the possibility that other emotions, some of which look decidedly non-basic –contempt, shame, guilt embarrassment, and awe– might find their way onto the basic list (Ekman, 1992, p. 192). Indeed, Ekman (1999, p. 57) went so far as to allow that, in the end, it might turn out all emotions are basic. Yet, if so, the basic/non-basic distinction would collapse.

Affective scientists are beginning to actively explore this possibility. In this regard, even Colombetti (2014, p. 40) acknowledges that what she regards as a BET-induced negative trend “is changing, although slowly.” Increasingly, emotion scientists are going the right way – they are appropriately widening their focus beyond an allegedly arbitrary subdivision of emotions. And, if it that were not already the case, it could and should be so. Either way, if there is any legitimate worry about BET it can’t be that it has intrinsically and irredeemably bad methodological effects on emotion research.

The true objection to BET must be –more in line with Hufendiek (2016) – that once affective science widens its gaze it will be discovered that BET is false. However, as already noted, at this stage of the game that, of course, is just an empirical bet.

So, is this a bet that BET is, in fact, losing as each empirical hand is revealed – as Hufendiek (2016) imagines? Is it the case –to rehearse Hufendiek’s (2016, p. 66) reasoning once again– that because “research in this field is still very young” we can expect evidence that many, if not all, emotions have affect program features? Let us assume that the evidence that canonical basic emotions have basic profiles continues to amass. Indeed, let us go further and assume –just as Hufendiek (2016) projects– that, in the end, it turns out all so-called non-basic emotions have a basic profile.

In such a case would we have sufficient grounds for rejecting BET? No. The reason is simple. To reject BET on these grounds depends on characterizing its core distinction as a distinction between two mutually exclusive classes of emotions that have exactly contrasting properties which do not overlap, as Colombetti (2014), Hufendiek (2016), and Caruana (2017) assume BET does. As far as we are aware, nowhere do proponents of BET insist on casting the basic/non-basic distinction in such terms. Notably, BET advocates have little to say about the positive characteristics of non-basic emotions, and tend only to characterize them in terms their *not being basic* (see, e.g., Griffiths, 1997). Griffiths (2003) does better in speaking of non-basic emotions as those that mediate social interactions and which can figure in folk psychological narratives. As he repeatedly emphasizes, it is emotions of this kind that are of particular interest to philosophers. Yet, so construed, there is nothing about non-basic emotions that would preclude them from sharing many of the characteristic features of basic emotions.

Importantly, it would not matter even if BET advocates did or do embrace the basic/non-basic emotion distinction in the way its critics characterize that distinction – for nothing would oblige defenders of BET to do so. Put otherwise, the basic/non-basic emotion distinction need not be drawn in terms of two kinds of emotions that have exactly contrasting, mutually exclusive features. The distinction can be drawn in other, more sophisticated ways – in ways that do not carve up the logical space so that basic emotions, *and only* basic emotions, have an evolutionary basis and exhibit distinctive affect program features.

Differentiating basic and non-basic categories will be viable just in case there is a single identifiable feature that can be ascribed to the one category but not to the other. For this reason, it is possible to have a crisp, clean and clear distinction between basic and non-basic emotions that does not preclude that a great many of the features of basic and non-basic emotions might overlap.

To see this point clearly, consider by way of analogy *Coca-Cola Classic* and *Coca-Cola Zero.* Both are kinds of Coca-Cola but they are not the same kind of Coca-Cola. Yet the two kinds of Coca-Cola are basically the same; they share all the same ingredients, in the same mix and portion, apart from sugar. Coca-Cola Classic has sugar, while Coca-Cola Zero has none – hence it has no calories. Thus, the two kinds of Coca-Cola can be cleanly and clearly distinguished by testing for the presence or absence of sugar; but testing them on the basis of any of their other, overlapping features would not enable us to discern to which kinds any particular instances belong.

There are major advantages of drawing the basic/non-basic emotion distinction in terms of non-basic emotions having an additional ingredient rather than mutually contrasting properties. For example, it avoids committing BET to the implausible idea that non-basic emotions must be “human-specific higher cognitive reaction[s] without any roots in the evolutionary history of other species” (Hufendiek, 2016, p. 66). On the extra ingredient reading of the basic/non-basic emotion distinction, BET can hold both that basic emotions have homologues in other species, and their origins in our phylogenetic ancestors, while also holding that non-basic emotions possess such features. We agree with Hufendiek (2016, p. 66) that it is plausible that non-basic emotions will have “an evolutionary basis that only develops further and changes or broadens its function in human life.” Yet that is perfectly compatible with an extra-ingredient way of carving out the basic/non-basic distinction.

We can now return to consider an idealized version of Hufendiek’s (2016) argument against BET. What if investigation into the emotions should reveal — as Hufendiek (2016, p. 67) anticipates — that there are “only very vague and gradual” features to differentiate between basic and non-basic emotions? Would emotion researchers need to reject the basic and non-basic distinction and instead embrace only a “differentiated theory of emotions” (Hufendiek, 2016, p. 68)? In such a case, wouldn’t affective science be better off classifying emotions “along a gradual spectrum, where some might be evolutionarily older, or more hardwired, or less sensitive to social context than others” (Hufendiek, 2016, p. 68)?

Not necessarily. Certainly, there is no forced choice here. Just because we can order emotions along a continuum – of, say, their evolutionary age, or susceptibility to change under social influences – does not preclude our drawing a firm and scientifically tractable distinction between basic and non-basic emotions.

In sum, the fundamental error with Colombetti’s (2014) and Hufendiek’s (2016) arguments, even in their idealized forms, is that both assume that BET is or must be committed to the idea that for any characteristic feature of basic emotions, a non-basic emotion will have the correspondingly opposite features. Yet, to echo Colombetti (2014), there is ‘no clear rationale’ for thinking that BET has only one way to carve out the basic/non-basic distinction. What is truly ‘arbitrary’ is to assume that there is only one possible characterisation of that distinction that could capture ‘the’ distinction that BET requires.

If this analysis is correct, then, *pace* Hufendiek, even if all emotions turn out to have affect program features, this would not suffice to show the basic/non-basic emotion distinction to be otiose or misguided. It would not show that the basic/non-basic emotion distinction is problematic, too strict or conceptually unsound. It would only show that we need to draw that distinction in a different way.

## Reasons to Bet on Bet

If the defanging and defusing efforts of the previous section succeed, then BET – as construed under the extra-ingredient rendering of the basic/non-basic distinction – may well still be a good bet. Is it?

Do any independent reasons motivate adopting the extra-ingredient account of non-basic emotions? Yes. Philosophers have long argued that emotions should be identified with certain kinds of contentful attitudes. This is because they hold that contents play defining and individuating roles that determine if an emotion is in play and which emotion is in play. Moreover, emotions have a normative dimension that can only be accounted for if they are assumed to be some kind of contentful attitude.

According to this content-based view of the emotions, having certain feelings of a characteristic sort do not suffice for having an emotion. Consider a case in which you feel insulted by a perceived slight. That feeling only suffices for your being emotionally upset, say angry, as long as you believe that you have been insulted. If that same feeling were to persist after you discover, and come to believe, that you were not insulted, then ascribing anger to you would no longer make sense. The feeling would not connect with the rest of your contentful attitudes in the right way. Hence, it would not count as an emotion at all.

Emotions so conceived are assumed to be or necessarily involve content-bearing attitudes of some kind. Thus, emotions are thought to be or embed contentful attitudes – it is their content that makes them, by their very nature, “rationally assessable and reason-sensitive” (Morag, 2016, p. 152). Emotions proper, so the familiar story goes, must have appropriate kinds of contents – be they contentful appraisals, evaluations, construals or judgments – if they are to stand in the right kinds of normatively constrained relations to other contentful attitudes^10^.

On this sort of content-based view of the emotions, something qualifies as an emotion only if it has the appropriate content. This has an unfortunate consequence if we stick with the policy of distinguishing basic and non-basic emotions in terms of mutually exclusive properties. For if it were assumed that affect programs lack contentful properties then emotions that fall into the so-called basic emotions won’t count as emotions at all.

On the content-based view of emotions, having content is *the essential* ingredient, not just *an extra* ingredient. There is an exact parallel with the way some people view Coca-Cola Classic and Coca-Cola Zero. They regard only the former to be a kind of Coca-Cola. Similarly, proponents of the content-based view of emotion hold that any so-called emotion that lacks content just isn’t the real thing. Following Griffiths (2003), let’s call this view the Philosopher’s Favorite.

That is one way to go. Another would be to press for the opposite conclusion – holding that so-called basic emotions are the only real emotions. One might be inclined to such a view, because, say, on scientific grounds we think that only basic emotions pick out a natural kind (Griffiths, 1997). Calling on our analogy once again, that would be parallel to insisting that the only real Coca-Cola is Coca-Cola Zero, because, say, it is more elemental and chemically fundamental. Let’s call this view the Scientist’s Favorite.

It would be a tough business to decide between such warring Philosophical and Scientific criteria. But, thankfully, we needn’t try. We can reasonably doubt the extreme view that emotions are, as a class, always and everywhere defined by, and thus necessarily entail, content. Yet we can also accept that basic emotions pick out real emotions without veering to the opposite extreme of denying the existence of non-basic emotions with contentful characters, or denying that their contents partly define such emotions.

The point is that we have reason to take a modestly pitched extra-ingredient account of the basic/non-basic emotion distinction seriously if it is assumed that at least some emotions have a contentful character that cannot be accounted for in terms of their affect program features. Crucially, the extra-ingredient rendering of the basic/non-basic distinction allows us to walk a middle path between two less plausible, more extreme views of the emotions – those that in different ways propose collapsing all of their essential features into a single set.

So far, so good. Yet, by admitting that some emotions have an extra ingredient that distinguishes them from other emotions, we are obliged to explain how that extra ingredient is acquired and how we should think about how basic and non-basic aspects relate in cases of emotions that have both elements.

Colombetti (2014) cites the failure of existing accounts of this relation as “another reason to drop the notion of basic emotions” (p. 46, see also Ortony and Turner, 1990). She is surely right that giving up the distinction would spare us the trouble of having to develop a better, alternative account. As she puts it: “Getting rid of the notion of basic emotions would also eliminate the need to explain how alleged basic emotions relate to non-basic ones” (p. 40). We concur that the standard options are inadequate, especially if the aim is to account for the contentful dimension of emotions. But, as we shall show, the sorts of accounts she considers do not exhaust all available options.

The most familiar accounts of how basic emotions might relate to non-basic emotions and might potentially explain their emergence are building block and blending theories. The common denominator in both of these kinds of theories is the assumption that non-basic emotions are somehow comprised of, or constructed out of, basic emotions. A blending theory, the more popular of the two types of theory, imagines basic emotions to be like primary colors, forming a kind of emotion palette from which to form non-basic emotions (Plutchik, 2001). In the simplest versions, such a theory might tell us that awe is a mix of fear and surprise; that guilt is a mix of joy and fear; that love is a mix of joy and acceptance. Colombetti (2014) worries that in general non-basic emotions require something more than mere mixtures of basic emotions. She gives examples of emotions that require having certain kinds of thoughts –such as love and guilt– that reveal pure blending theories to be insufficient (see Colombetti, 2014, p. 42). The same worry applies, *mutatis mutandis*, to building block theories. By these lights, an extra ingredient is needed if we are to get non-basic emotions from basic ones – an ingredient that cannot be accounted for by either pure building block or blending theories.

On this analysis, neither pure building block nor blending theories of basic emotions suffice – neither have credible resources to explain how, through their mere combination, contentless emotions can generate contentful emotions. Prinz (2004) advances an impure, more complex version of blending theory – adding a crucial extra dimension to his explanation. He proposes that basic emotions may need to be calibrated with other, culturally-sourced cognitive states that are not emotions in order for there to be non-basic emotions. He gives the example of schadenfreude, which he proposes is a feeling of joy triggered by the thought of someone suffering (Prinz, 2004, p. 147–148). Crucially, schadenfreude is not, for Prinz (2004), merely a blend of the feeling of joy and the additional thought that someone is suffering. Rather on his model, schadenfreude is a feeling of joy that has been triggered in a special way – by being filtered through contentful attitudes – and is thus calibrated with someone’s suffering. Accordingly, cognitive contributions, beyond those that can be provided by building or blending base emotional elements, are necessary for having non-basic emotions.

*Prima facie*, a calibration theory of this sort looks more promising than its purist rivals. However, it cannot fully explain how non-basic emotions arise from basic ones unless it adequately explains – rather than simply presupposes – the genesis of the culturally sourced, cognitive contents that it relies on to do the relevant, additional work. Arguably, existing accounts fall short on just this requirement. For example, both Prinz (2004) and Hufendiek (2016) rely on classical teleosemantic theories to account for the contents that they assume feature in emotional episodes. Classical teleosemantic theories of content seek to explain representational contents in terms of biological functions (Millikan, 1984, 2005; Papineau, 1987). Yet, though promising, all such theories face well-known, crippling problems – principally, it is argued that there is “a root mismatch between representational error and failure of biological function” (Burge, 2010, p. 301, for a full discussion see Hutto and Myin, 2013, Ch. 4, Hutto and Myin, 2017).

Luckily, there is another way to explain the natural origins of the contents that are needed to augment basic emotions such that there can be non-basic emotions. Attempts are underway to try to explain how contentful forms of cognition first came on the scene, in phylogeny, and now come on the scene, in ontogeny, by appeal to socio-cultural scaffolding (Hutto and Satne, 2015). If this proves possible then a complete story can be told, without gaps, of how we move from basic to non-basic emotions and how these might inter-relate (Hutto and Myin, 2017). To tell this story in detail would involve specifying how basic emotional episodes come to inspire and feature in “complex emotional episodes that figure in folk-psychological narratives about mental life” (Griffiths, 2003; see also Goldie, 2000, 2003, 2012; Hutto, 2008).

A virtue of this the socio-cultural scaffolding theory is that it accounts for the extra ingredient – the content– needed for there to be non-basic emotions in a way that pure building block and blending theories cannot. Beyond that, this duplex account of emotions allows for non-basic emotions to have basic roots in phylogenetic, ontogenetic histories – roots that serve as platforms that explain how the emotion-relevant concepts and contents needed for non-basic emotions arise.

## Adjusting Our Bet

For all we’ve said, some readers may harbor some residual concerns about understanding basic emotions in terms of affect programs. To think of basic emotions as affect programs is to regard them as evolutionary adaptations that are involuntarily and automatically triggered and which result in a sequence of responses – these patterns are what exhibit the characteristic profile for each basic emotion (Ekman, 1992; Griffiths, 2004).

Does thinking of basic emotions as affect programs also imply that they are necessarily brain-bound, going against the burgeoning 4E trend in cognitive science (see, e.g. Newen et al., 2018)? A number of expositors of BET certainly present it as if it is committed to the idea that affect programs are neurally housed. Caruana (2017, p. 87) holds that proponents of BET “argue that emotions are discrete mental entities localized in the brain in the form of affect programs.” Colombetti (2014) thinks of BET’s basic emotions as products of “genetically determined sets of instructions called *affect programs* which once activated generated a series of distinctive changes” (p. 26, emphasis original). And, with reference to his 2003 book, Hufendiek (2016) tells us that “Ekman … claims that affect programs are *literally inscribed* into our neural circuits” (p. 68, emphasis added).

We find no need for BET to make such a commitment to brainbound programs. Nothing in the very idea of affect programs entails such brainboundedness. Indeed, if anything, official statements about BET only make much weaker claims about the brain’s involvement in affect programs^11^. To quote Ekman (2003) at length:

Affect programs are, like the emotion databases, *a metaphor*, for *I do not think there is anything like a computer program sitting in the brain*, nor do I mean to imply that only one area of the brain directs emotion. We know already that many areas of the brain are involved in generating emotional behavior, but until we learn more about the brain and emotion, a metaphor can serve us well in understanding our emotions (p. 66, emphases added).

In any case, even if some proponents of BET do think of affect programs as brain-bound sets of instructions, they needn’t do so^12^. There is nothing in the very idea of BET that necessitates such a claim. Thus we need not dispense with BET or the idea of affect programs in order to view emotions as “dynamically organized patterns that are realized by the whole organism” (Hufendiek, 2016, p. 69). We can do this, for example, by updating our understanding of an affect program, incorporating it within a radical enactivist account of emotion. This would be, as Caruana (2017, p. 100) recognizes, to offer an account of basic emotions that emphasizes not the body as such but the acting body; one that portrays emotional episodes as enacted. Basic emotions can thus be understood under the auspices of a radical enactivism that emphasizes the full-bodied, active capacities of living organisms in the generation of affective phenomena (see Hutto, 2012).

Still, there may be a lingering worry. Wouldn’t construing affect programs by the lights of radical enactivism lead us into the arms of an unworkable behaviorism?

This is a general, much-voiced worry about radical enactivism – or indeed, any non-representational account of cognition. With direct reference to radical enactivism, O’Brien and Opie (2015) put the worry this way:

there is a fundamental problem with the idea of contentless intentionality: it’s been tried before, and it doesn’t work. Back then the scheme was known as ‘behaviorism,’ rather than ‘targeted directedness,’ but the two ideas are of a piece. Behaviorists sought to explain animal behavior, including all the complexities of human problem solving and language, in terms of the history of stimulus–response events to which organisms (of each kind) are typically exposed. The bankruptcy of this approach consists in the fact that moment-by-moment stimuli are simply too impoverished to account for the richness, variety, and specificity of the behaviors that animals exhibit. It just isn’t possible to explain the ability of evolved creatures to selectively engage with features of the environment—in other words, engage in targeted behavior—without supposing they employ internal states that in some way represent those features (p. 724, see also Adams, 2018, p. 23).

Employing this logic, if affect programs are not brain-bound representational-cum-computational instructions, or driven by such, then we have no choice but to understand them only using the resources of stimulus-response behaviorism.

Applied to the question of basic emotions, the worry is that radical enactivism, in negatively eschewing mental representations, offers nothing by way of positive explanatory resources other than those offered by a crude behaviorism^13^. Hufendiek (2016) makes this specific version of the objection explicit, proposing that our preferred non-representationalist enactivist theory of emotion is:

subject to the most basic objection that cognitivism has brought forward, that a characterization of emotions *as mere bodily reactions and behaviors* completely misses the normative dimension of emotions. Fear reactions are not *just automatic responses to a given stimulus*. They are reactions that can be adequate or inadequate depending on whether the situation in question is really dangerous or not (Hufendiek, 2016, p. 96, emphases added).

Driving home the point, Hufendiek (2016) offers the following illustration:

If we try to characterize fear as a reaction that is reliably caused by the presence of certain dangerous stimuli that under normal conditions cause typical bodily reactions (such as the heart beating faster, adrenaline being released, and so on) and finally think of the whole process as a mechanism created by evolution, we end up with a crude and simplistic behaviorist model of emotions (Hufendiek, 2016, p. 96).

*Pace* Hufendiek, radical enactivism isn’t just a different label for a crude stimulus-response behaviorism^14^. Perhaps the easiest way to see this is to note that radical enactivism embraces a notion of embodied activity that does not reduce to the behaviorist notion of a response to a stimulus. What’s the difference? Importantly, as Jacob (2015) observes, enactivists break with the behaviorism in setting their face against the idea that embodied activity should be understood as any kind of simple output in reply to an input. Indeed, enactivists typically reject the input-output model of mind outright (see, Villalobos and Dewhurst, 2017a,b).

In promoting the idea that mentality is fundamentally wide reaching and dynamical in character, radical enactivists abandon both the linearity and instrumentality assumptions that exemplify the vision of mind promoted by their behaviorist and cognitivist rivals. The linearity assumption holds that cognitive processes follow a standard sequence: “information flows from the outside world in, through the sensory systems to perception, cognition, the motor system, and then out again into the world” (Jacob, 2015, p. 227). The instrumentality assumption holds that action stands in a means-end, as opposed to constitutive, relation to cognition: cognition is the means by which actions are produced or generated.

Crude behaviorism, like radical enactivism, rejects the instrumentality assumption. However, radical enactivism fundamentally disagrees with any behaviorism that subscribes to the linearity assumption. Radical enactivists certainly do not think that the embodied activity which they take to constitute basic emoting reduces to responses that are triggered by a specific stimulus and which unfold in a mechanical, script-like way.

The cornerstone, big idea of radical enactivism upon which its other positive proposals about basic and non-basic minds rest is that “the embodied activity of living beings provides the right model for understanding minds” (Hutto and Myin, 2013, p. 4)^15^. Pivotially, radical enactivism focuses on living creatures and systems not dead mechanisms when it comes to modeling and understanding minds (see also Kirchhoff and Robertson, 2018)^16^.

For this reason, the notion of embodied responsiveness that radical enactivism promotes cannot be adequately understood in terms of simple, blind mechanisms that are incapable of any kind of novel adjustment to circumstance. The kind of embodied activity that enactivists think typifies basic emoting therefore does not subscribe to an empiricism or associationism that conceives of habits “atomistically and as automatisms” (Barandiaran and Di Paolo, 2014, p. 1). Radical enactivists thus ally themselves to what Barandiaran and Di Paolo (2014) dub organicism – an idea that denotes a more venerable trend of thought about the nature of embodied activity and habits. It is an idea that has yet to be, “taken up by mainstream cognitive neuroscience and psychology. Habits, in this tradition, are seen as ecological, self-organizing structures that relate to a web of predispositions and plastic dependencies both in the agent and in the environment” (Barandiaran and Di Paolo, p. 1).

Crucially, in modeling minds on living systems and not inanimate mechanisms, enactivists propose to enliven our picture of embodied habits—proposing that even in the absence of cognitive contents our responsiveness is not lifeless and mechanical. Accordingly, it regards all forms of cognition –even the most basic variety– as precariously open to the particulars of situations and, hence, capable of novelty. Radical enactivism characterizes embodied activity that constitutes basic cognition as a kind of context-sensitive responsiveness which is flexible and open to the unique features of situations. It does not understand such mentality in terms of slavishly scripted, reflex-like responses to a stimuli, executed by blind or automatic mechanisms.

Why does this matter? It exposes that the dilemma Hufendiek (2016) poses for radical enactivism presents us not with a terrible, inevitable choice but a false choice. It is demanded that we either accept cognitivism or embrace an unworkable behaviorism. We must either accept that cognition is a matter of contentfully representing and modeling the world –namely, that there is ‘intel inside’ driving embodied activity that qualifies as cognitive– or be condemned to explaining mindedness in terms of inflexible automatic reflexes that are thoughtless, blind, unthinking, mechanical, script-like and lacking in all novelty.

To see why Hufendiek’s choice is in fact a false choice, it helps to give attention to infamous story of the Sphex wasp and the way that story has been used in the cognitive sciences. This is the story of Sphex, as told by Wooldridge (1963):

When the time comes for egg laying, the wasp Sphex builds a burrow for the purpose and seeks a cricket which she stings in such a way as to paralyze but not kill it. She drags the cricket into the burrow, lays her eggs alongside, closes the burrow, then flies away, never to return. In due course, the eggs hatch and the wasp grubs feed off the paralyzed cricket, which has not decayed, having been kept in the wasp equivalent of a deepfreeze. To the human mind, such an elaborately organized and seemingly purposeful routine conveys a convincing flavor of logic and thoughtfulness—until more details are examined. For example, the wasp’s routine is to bring the paralyzed cricket to the burrow, leave it on the threshold, go inside to see that all is well, emerge, and then drag the cricket in. If, while the wasp is inside making her preliminary inspection, the cricket is moved a few inches away, the wasp, on emerging from the burrow, will bring the cricket back to the threshold, but not inside, and will then repeat the preparatory procedure of entering the burrow to see that everything is all right. If again the cricket is removed a few inches while the wasp is inside, once again the wasp will move the cricket up to the threshold and re-enter the burrow for a final check. The wasp never thinks of pulling the cricket straight in. On one occasion this procedure was repeated 40 times, always with the same result (pp. 82–83).

Keijzer (2012) provides a detailed and highly revealing analysis of this oft-repeated Sphex narrative, showing its illicit use in promoting the view that these wasps –and, by association, insects more generally– never modify their routine behavior. The story of Sphex has worked to convince many of the primary message that “insect intelligence is very basic and mechanical” (Keijzer, 2012, p. 504).

The Sphex story has done much to promote the idea that a basic mind simply equates to a blind mechanism. By such lights, basic minds are precisely the sort of minds that can be adequately accounted for in stimulus-response terms, or by positing simple computational devices, those that generate a strict and rigid sequence of outputs in response to triggering inputs ^17^.

Importantly, the simple, diverting power of characterizing Sphex as inflexible mechanism flies in the face of the empirical facts about the nature of its waspish intelligence. These facts, though debated, have been known for some time. Indeed, to some extent they appear to have been known from the start, even though a great deal of further research was required in order for a truly balanced account of the details to emerge.

Over time, questions have been raised about whether all species of Sphex wasp, let alone all insects, are really condemned to behavioral fixity. Ethologists have long investigated to what extent the repertoires of such creatures admit of some degree of behavioral variability.

As things stand now, the facts of the matter present us with an interesting and complicated picture: the behavior of Sphex wasps and other insects is mixed and equivocal. Their behavior is neither slavishly fixed nor entirely variable – it’s a bit of both. How these animals behave depends on many factors, including the species of wasp in question and their particular habitat. It turns out that details of their embodiment, situation and circumstances matter. In the end, the correct answer appears to be that, “these wasps can initiate new kinds of behavior under the proper circumstance” (Keijzer, 2012, p. 414)

The parable of Sphex is important because it highlights two things. Firstly, it beautifully illustrates the advantage of characterizing basic minds in radical enactivist terms, where such minds are modeled on living systems and not dead mechanisms. For the aforementioned reasons, doing so enables us to attend better to the full set and true character of the empirical findings. Thus, adopting an enactivist perspective allows us to better capture the nature of phenomena under scrutiny than does using the theoretical machinery of its mechanistic rivals.

Secondly, we should learn from the story about how the Sphex story has been used, or rather misused, in the philosophy and sciences of the mind. What is deeply concerning is “the endless repetition of humans retelling the story as a matter of significance *despite all the available counterevidence*” (Keijzer, 2012, p. 515, emphasis added). The use of the Sphex case in the field offers up a salient methodological moral. The whole affair gives us pause to reflect: “One may wonder here whether cognitive issues should be settled by finding proper examples that fit existing theories, or whether theories should be developed that deal adequately and systematically with the findings that nature provides” (Keijzer, 2012, p. 516).

This is a reminder that philosophers may be better served to operate with a policy of looking first before rushing to theorize (Hutto and Satne, 2017, 2018). Such an approach enables us to come at the empirical data in a new and better ways before making any attempt try to craft explanations or test specific hypotheses. This is important because the explanatory adequacy of any theory or hypothesis – our explanation of the facts – will in large part hinge on the empirical adequacy of how we frame or describe those facts, as the Sphex story so clearly illustrates.

In general, we might be suspicious of the idea that philosophers are in the first-order business of producing scientific theories and explanations in any case. Philosophical frameworks are not scientific theories. Nor do they immediately generate testable models, even if they are answerable to empirical findings and can inspire and help to direct productive programs of empirical research.

Functionalism, as its father Putnam told us back in 1967, principally amounts to “the putting forward, not of detailed scientifically ‘finished’ hypotheses, but of schemata for hypotheses” (Putnam, 1967/1992, p. 54). More recently, in reflecting on the extended mind thesis, Clark (2011) reminds us that some important philosophical issues are unlikely to admit of straightforward scientific resolution even though they are “scientifically important, and able to be scientifically informed” (Clark, 2011, p. 454).

We have so far shown that radical enactivism does not reduce to a crude behaviorism. It has richer resources than the latter and offers a very different account of BET’s affect programs than does its mechanistic rivals. Even so it may be thought that there is still a crippling problem with our preferred account of basic emotions – one that cannot be so easily addressed. In nutshell, the worry is this: because radical enactivism gives up on the idea of contentful mental representations its take on basic emotions is unable to account for their normative dimension. How, so the familiar objection goes, can an account of the emotions that eschews representationalism and the idea that basic emotions are contentful possibly account for or accommodate their normative aspects?

We do not deny that basic emotions exhibit a kind of normativity. Yet before trying to explain how this can be so on a radically enactive account we need to get clearer about what kind of normativity basic emotions actually exhibit. To do that we need to get beyond the familiar philosophical penchant for over-intellectualizing any phenomenon under scrutiny^18^.

Following Moran (2002/2017), we can take loving and caring to be exemplars of basic emotions and understand them as “essentially active responses to something else, and answerable to the specific norms of that something else” (p. 153). Such norms are objective, they are not up to the individual. Speaking of the norms of pleasure, for example, Moran (2002/2017) explicates this point by reminding us that “idly stroking some surface will determine that only some activities and only some surfaces will be possible providers of just those pleasures” (p. 153). Hence, there are conditions of satisfaction for such norm-guided activity but, crucially, those conditions need not be contentfully represented, as intellectualism would have it.

Moran (2002/2017) defends this brand of anti-intellectualism by saying: “None of this is to say that all these different types of response should be seen as simply forms of judgments” (p. 153). Few today would defend the view that basic emotions are judgments, *stricto sensu*. Many are however inclined to think they must be representational in some sense, ala Hufendiek, if we are to account for their normative dimension. Yet, if we follow Moran’s logic, there is no reason to think basic emotional responses need to involve contentful mental representation of any sort. It is easy to see why if we consider that contentful mental representations are just the analog, or replacement notion, for explicit judgments – differing only in that they are implicit and out of sight, operating at the so-called sub-personal level. The pivotal point is that if contentful judgments are superfluous for doing the required explanatory work and hence otiose for understanding the kind of normativity that basic emotions exhibit, then contentful mental representations are superfluous and otiose for the very same reason.

In sum, it appears it is possible to adjust and update our understanding of affect programs in radical enactivist terms without embracing either cognitivism or behaviorism. In doing so, we propose a new better BET. This new BET, we think, is a pretty good bet.

## Conclusion

By clarifying the possible ways of drawing the basic/non-basic emotion distinction, we have defended BET by defusing some allegedly crippling objections against it. We have shown why those objections fail, even when they are advanced in their ideal forms. We have also shown that there is a plausible way to understand how basic emotions might give rise to, and relate to, non-basic emotions – once we conceive of the basic/non-basic emotion distinction as one in which non-basic emotions are distinguished by an extra ingredient. Finally, we have shown that the affect programs that BET relies on can, in principle, be understood through the lens of a radical enactivism, such that they need not be conceived of as brain-bound instructions nor mere stimulus-response patterns.

## Author Contributions

The paper was a collaborative, joint effort in which DH took the theoretical lead. IR conducted primary research into the subject matter and was engaged in every stage of the co-authoring. MK played a more supportive and advisory role. All co-authors conducted research on this paper as part of their joint ARC Discovery Project - Mind in Skilled Performance.

## Conflict of Interest Statement

The authors declare that the research was conducted in the absence of any commercial or financial relationships that could be construed as a potential conflict of interest.

## Footnotes

1 The defining characteristic of basic emotions, on this theory, is that they have evolved because of their adaptive value in enabling organisms to navigate “fundamental life-tasks” (Ekman, 1992, p. 196; see also Griffiths, 1997).

2 DeLancey (2002, p. 3) usefully characterizes BET’s driving assumption as a commitment to the idea that some emotions are biologically based, pancultural syndromes, understood as “coordinated collections of complex biological responses that occur together.”

3 For example, a good deal of work has been devoted to investigating the neurobiological profile of the alleged basic emotions (see Vytal and Hamann, 2010 for a review; see also Wager et al., 2015; Clark-Polner et al., 2016; Nummenmaa and Saarimäki, 2017; Saarimäki et al., 2018). Despite this, there is a respectable degree of caution in the field about whether and to what extent neuroimaging evidence from, e.g., fMRI and PET studies, does or even could lend support to BET (Celeghin et al., 2017; Caruana, 2017; McCaffrey, unpublished).

4 Others also aim to defend BET, but they propose to do so in a different way than we do, and in response to different sorts of objections. Psychological constructionists, such as Barrett (2006) and Barrett and Russell (2015), have argued that BET is empirically unsound. They hold that neuroscientific and psychophysiological data do not support the hypothesis of original BET that there are one-to-one correspondences between specific emotion categories and profiles characteristic of affect programs. They take this to empirically falsify BET. By way of reply, Scarantino (2015, p. 344) has proposed revising and updating BET in a way that acknowledges “the constructionist critique while preserving the notion that basic emotions are specialized, evolved programs for dealing with fundamental life tasks.” Evaluating whether such an approach succeeds in dealing with the criticisms raised by constructionists falls beyond the scope of this paper. Our proposal for a new, better BET has a different purpose – it seeks to demonstrate that there is a logically coherent and empirically sound way to draw the basic emotion/non-basic emotion distinction that avoids the philosophical objections described above. Our aim is to clarify and defend BET, on philosophical grounds, in a way that has not been done hitherto.

5 We make no claim that Ekman and Friesen’s list definitively individuates six distinct basic emotions, each of which is made manifest via a unique affect program profile. Indeed, Ekman and Friesen (1971 p. 128) note that their participants were seemingly unable to reliably distinguish between fear and surprise. This would seemingly undermine the notion that Ekman and Friesen’s (1971) facial recognition data grounds a distinction between fear and surprise as basic emotions.

6 It is often argued that the BET research program should be expanded so as to take into account a wider range of emotions. Keltner and Cordaro (2017), for example, argue that the multimodal and highly intricate nature of emotional expression lends support to the contention that there may be far more basic emotions than Ekman and Friesen’s (1971) original big six. In his later writings Ekman (1999, p. 194) was expressly open to this possibility: he proposed that “the list of basic emotions and possible basic emotions is not a short one.”

7 Others have made similar claims about the bad consequences of BET. Ortony and Turner (1990, p. 315) observe that BET has resulted in widespread acceptance “that there exists a small set of basic emotions.” Frijda and Parrott (2011, p. 207) also contend that “the construct of basic emotions has promoted undesirable consequences: a focus on a small subset of human emotions.” Celeghin et al. (2017, p. 2) go further and describe BET as being committed to “a view that purports the existence of a small number of so-called basic emotions.” In a tentative tone, Hufendiek (2016) raises a similar worry about BET. She writes, “A methodological problem with the postulation of basic emotions could be that there is a tendency in the research literature to postulate a fixed set of basic emotions extrapolated from one’s own research in a single field. This is exactly what Ekman does in postulating that there are six affect programs because this fits with his finding that there are six cross-culturally occurring facial expressions*”* (Hufendiek, 2016, p. 68). Here, Hufendiek uses the term affect program interchangeably with basic emotion, as is typical in the BET literature. It should be stressed again that Ekman did not postulate that there are a fixed set of basic emotions. He is open to the empirical possibility that all emotions exhibit a basic profile (Ekman, 1999, p. 57).

8 In her own words, “affective science would be better off dropping the notion of basic emotions” (Colombetti, 2014, p. 26, p. 40).

9 For example, she predicts that “it is likely that evidence for other stereotypical expressions of so-called higher cognitive emotions will be found” (Hufendiek, 2016, p. 66).

10 The attitudes might be thought to be explicitly formed propositional attitudes, such as beliefs or judgments. Or they might be thought to be some other kind of pre-reflective, implicit contentful attitudes (for a detailed discussion of this point see Morag, 2016, p. 31 ff.).

11 Some theorists, however, do make stronger and more metaphysically committed claims about affect programs. For example, Tomkins (1962/2008) thought of an affect program as “a subcortical structure which can instruct and control a variety of muscles and glands to respond with unique patterns of rate and duration of activity characteristic of a given affect” p. 135).

12 Although Ekman clearly rejects the idea that affect programs are literally neuronally instantiated computer programs, it is unclear whether he takes the realization base of basic emotions to be exclusively neural. Ekman (1992) claimed that “it is necessary to posit emotion-specific central nervous system (CNS) activity in my account of basic emotions. The distinctive features of each emotion, including the changes not just in expression but in memories, imagery, expectations and other cognitive activities, could not occur without CNS organization and direction” (Ekman, 1992, p. 182). This statement is neutral on the question of whether the extra-neural aspects of the CNS are thought to play a constitutive or a merely causal role in the realization of basic emotions. Enactivists do not fence sit on this matter: they characterize emotions as realized not just in the brain but across the brain, body and world though dynamical processes comprising organismic engagements with environmental features of specific situations (Hutto, 2012).

13 Notably, some have argued that the kind of unworkably crude behaviorism that critics, such as O’Brien and Opie (2015), Hufendeik (2016), and Adams (2018), have in mind does not accurately depict actual historical versions of behaviorism. Rather it is deemed to be a straw position - a familiar but misleading caricature of real behaviorism (see Barrett, 2016).

14 Even Westra (2018, p. 1) acknowledges that, “Despite its anti-representationalist bent, REC is not wholly behaviouristic.”

15 Bearing this in mind, it is odd that Baggs (2017) criticizes radical enactivism for advancing a purely negative, representation-bashing agenda. By his lights, radical enactivism only takes and never gives: It allegedly lacks a core set of ideas around which a positive project could be built. Baggs’s (2017, p. 2) failure to notice radical enactivism’s commitment to the life/mind model may in part explain why he finds it “hard to imagine how it could be translated into a positive empirical program.” Thompson (2018) echoes this sentiment, arguing that a successful enactivism should concern itself with basic theoretical and empirical questions and use these “to motivate the careful construction of a positive theoretical framework with testable models”. As a criticism of radical enactivism, the complaint that the framework offers nothing positive is ironic given that foundationally both autopoietic and radical enactivism “emphasize the importance of active, embodied, environmentally situated engagements for our understanding of organisms and their mentality; and both view understanding developmental processes as necessary to the understanding of life and mind” (Kee, 2018, p. 6). Where the two enactivisms part company is over the question of whether sense-making is an indispensable and theoretically important construct (see Kee, 2018 and also Barandiaran, 2017 for discussions of this point).

16 Radical enactivism is studiously neutral with respect to strong metaphysical claims that are often associated with other forms of enactivism such as, the life/mind deep continuity thesis as defended by Kirchhoff and Froese (2017).

17 Keijzer (2012, p. 507) observes that thinking of basic minds or intelligence in this way leant itself to “a new type of reader, those working in fields like control theory, engineering neural circuits, and what eventually became the cognitive and computational neurosciences.”

18 As Moran (2002/2017) says, “the philosopher’s occupational tendency is to ‘intellectualize’ anything he can bring himself to take seriously in human life, to treat all internal conflicts as conflicts of judgments” (p. 149).
